# Incidence and Short‐Term Prognosis of Acute‐On‐Chronic Liver Failure Defined by Japanese Criteria: A Single‐Center Retrospective Cohort Study From Urban Japan

**DOI:** 10.1002/jgh3.70300

**Published:** 2025-11-10

**Authors:** Hidehiro Kamezaki, Terunao Iwanaga, Takahiro Maeda, Junichi Senoo, Sadahisa Ogasawara, Shingo Nakamoto, Naoya Kato

**Affiliations:** ^1^ Department of Gastroenterology Eastern Chiba Medical Center Togane Japan; ^2^ Department of Gastroenterology, Graduate School of Medicine Chiba University Chiba Japan

**Keywords:** acute‐on‐chronic liver failure, infections, liver cirrhosis

## Abstract

**Background and Aim:**

Acute‐on‐chronic liver failure (ACLF) carries high short‐term mortality, but real‐world evidence under the Japanese ACLF criteria remains limited. We assessed incidence, clinical profile, outcomes, and prognostic factors at a tertiary center in urban Japan.

**Methods:**

We retrospectively reviewed 363 consecutive hospitalizations of patients with cirrhosis (2014–2022) at a tertiary care center in Japan. ACLF per Japanese criteria was categorized as confirmed (meeting both PT INR/PT activity and bilirubin thresholds) or extended (meeting either biochemical criterion alone). We pre‐specified a parsimonious Cox model with age (per 10 years) and MELD‐Na (per 5 points); the primary outcome was time to all‐cause death within 90 days (administrative censoring at day 90).

**Results:**

ACLF occurred in 40/363 (11.0%) patients (confirmed *n* = 10, extended *n* = 30). Frequent precipitants were infection, gastrointestinal bleeding, and alcohol use, often in combination. The 90‐day mortality by Kaplan–Meier was 30.5%. Age (per 10 years) was associated with higher 90‐day mortality (HR, 2.29; 95% CI 1.31–4.00; *p* < 0.01), as was Model for End‐Stage Liver Disease including sodium (MELD‐Na) (per 5 points) (HR, 1.74; 95% CI 1.16–2.63; *p* < 0.01).

**Conclusions:**

In this Japanese single center cohort, ACLF (per national criteria) was not rare and carried substantial short‐term mortality. Age and MELD‐Na were dominant prognostic factors, underscoring early trigger control (notably infection, gastrointestinal bleeding, and alcohol cessation) and timely risk stratification in routine care.

## 1 Introduction

Acute‐on‐chronic liver failure (ACLF) is a complex syndrome characterized by the sudden deterioration of liver function in patients with preexisting chronic liver disease. This condition is associated with high mortality and morbidity and presents substantial clinical challenges due to its rapid progression and the need for intensive medical intervention [1, 2]. Pathophysiologically, ACLF is characterized by severe systemic inflammation, immune dysfunction, and multi‐organ failure, which contribute to poor outcomes and complicate its management [3].

While studies worldwide have explored various aspects of ACLF, it is important to recognize the distinct characteristics of ACLF within the Japanese ACLF criteria and compare them to those in other regions, such as Europe and Asia‐Pacific [4]. While the EASL‐CLIF definition restricts ACLF to patients with cirrhosis, [3] the APASL criteria include patients with chronic liver disease with or without cirrhosis and defines ACLF by an acute hepatic insult on chronic liver disease prior to ACLF onset [5]. This difference in diagnostic criteria can be explained by the varying backgrounds of the patients from these different geographical regions. In patients from Europe and the United States, alcoholic cirrhosis is prevalent, while in patients from Asian countries, acute exacerbation of chronic liver disease is commonly caused by hepatitis B virus. This long‐recognized regional pattern helps explain differences among APASL, EASL, and Japanese diagnostic frameworks. However, in Japan, according to a recent pilot study, the underlying cause was most often alcoholic (54.1% of registered cases) and had clinical features similar to those in Europe and the United States [6]. In addition, the diagnostic criteria for acute liver failure in Japan highlight that in the absence of a clear decline in liver function before the onset of liver dysfunction, the condition should be treated as acute liver failure [7]. The uniformity of the diagnostic criteria for acute liver failure and ACLF in Japan led to the understanding that the etiology of ACLF in Japan should be limited to cirrhosis. In addition, decompensated grade C cirrhosis with a Child‐Pugh score ≥ 10 was considered to be a progression of chronic decompensation, and was therefore excluded from the ACLF criteria [7]. Accordingly, we interpret the present findings within the Japanese clinical context and diagnostic framework.

The criteria in Japan define ACLF as liver damage manifesting within 28 days, with severe liver dysfunction in patients with compensated or decompensated cirrhosis and aggravating factors such as heavy alcohol consumption or infection. Furthermore, the criteria characterize liver dysfunction as having a prothrombin time‐international normalized ratio (PT‐INR) ≥ 1.5 or PT activity ≤ 40%, and total bilirubin (T‐Bil) concentrations ≥ 5.0 mg/dL (≈85.5 μmol/L).

Managing ACLF is inherently challenging because of the unpredictable nature of its progression and the variability in patient response to treatment. The prognosis of ACLF can vary significantly based on the underlying cause of liver disease, severity of liver dysfunction, and presence of extrahepatic organ failure [8]. Traditional prognostic models often fail to capture the dynamic changes occurring in ACLF, making it difficult to predict outcomes accurately [9]. This uncertainty complicates clinical decision‐making and highlights the need for more refined prognostic tools and treatment strategies.

To improve the understanding and management of ACLF, it is crucial to gather real‐world data which accurately represents the patient population and clinical scenarios encountered in routine medical practice. Real‐world data can provide insights into the natural history of ACLF, the effectiveness of different treatment modalities, and the effects of different prognostic factors in a more diverse and representative patient cohort [10]. Such data can help bridge the gap between clinical research and practice, leading to more effective and personalized treatment approaches.

However, many studies on ACLF are constrained by methodological limitations, such as patient selection bias, small sample sizes, and controlled environments which do not reflect the complexities of typical clinical settings. These limitations hinder the generalizability of the findings and their applicability to broader patient populations [11, 12]. For example, the controlled settings of clinical trials often exclude patients with comorbidities or varying degrees of disease severity, which limits the applicability of their findings to everyday clinical practice [13].

This study leveraged the unique setting of a tertiary medical center, the sole certified hepatology facility in its secondary medical care area, to minimize patient selection bias and provide a more accurate reflection of ACLF incidence and outcomes. The stable demographics of the region, which include a diverse patient population, offer an exceptional opportunity to study ACLF in a relatively unchanged population, thereby enhancing the reliability and validity of the data collected.

The hypothesis of this study was that examining ACLF in this stable demographic area, free from significant patient selection bias, would yield more accurate real‐world data. This information could enhance treatment strategies, inform policymaking, and ultimately enhance ACLF management. By focusing on a representative patient population in real‐world clinical settings, this study aimed to address the limitations of previous ACLF research and provide insights directly applicable to improving patient care. In Japan, even patients who show a marked abnormality in only one of the two key parameters—prothrombin time‐international normalized ratio (PT‐INR) ≥ 1.5/PT activity ≤ 40% or T‐Bil ≥ 5.0 mg/dL—have been reported to progress rapidly to multi‐organ failure within a short interval [4, 14, 15]. To capture this “pre‐ACLF” phase in routine practice, we therefore included an Extended‐ACLF category, defined as cases that meet either biochemical criterion alone, in our analysis.

Given prior evidence that age and MELD‐Na are among the most consistent short‐term prognosticators in ACLF, and to minimize overfitting in a cohort with limited events, we pre‐specified a parsimonious prognostic analysis focusing on these two covariates [2, 8, 9, 16, 17]. In the Japanese framework—where baseline Child–Pugh is restricted to 5–9—additional variability is limited and partly overlapping with MELD‐Na; therefore, Child–Pugh was not entered into the primary model [14].

## 2 Methods

### 2.1 Study Design

This study was a retrospective analysis of patients with liver cirrhosis who were hospitalized for any reason, not necessarily due to complications of cirrhosis.

### 2.2 Study Setting

This study was conducted at the study center, a tertiary care hospital in Chiba Prefecture, Japan. Chiba lies immediately east of Tokyo and is part of the highly urbanized Greater Tokyo metropolitan area. The hospital is certified by the Japan Society of Hepatology and serves as the principal hepatology center for its secondary medical‐care region.

### 2.3 Inclusion and Exclusion Criteria

Patients with liver cirrhosis who were hospitalized at the study center between April 2014 and December 2022 were included in this study. Patients who opted out of the study were excluded. No patients opted out; therefore, none were excluded for this reason.

### 2.4 Data Collection

Data were retrospectively collected from electronic health records and patient charts. ACLF cases were identified based on the diagnostic criteria for ACLF in Japan. Collected data included demographic information, clinical characteristics, treatment modalities, and patient outcomes.

### 2.5 Patient and Public Involvement

The present study was primarily based on a retrospective analysis of medical records data; thus, the involvement of patients or the public in the design, conduct, reporting, or dissemination plans of this research was neither appropriate nor feasible.

### 2.6 Parameters and Definitions

#### 2.6.1 Confirmed ACLF

ACLF was diagnosed when the patient had compensated or decompensated cirrhosis (Child‐Pugh score of 5 to 9) and presented with severe liver dysfunction within 28 days, with aggravating factors such as excessive alcohol consumption, infection, gastrointestinal bleeding, or exacerbation of the primary disease; PT‐INR ≥ 1.5 or PT activity ≤ 40%, and serum T‐Bil ≥ 5.0 mg/dL [4].

#### 2.6.2 Extended ACLF

Extended ACLF was diagnosed when any one of the following criteria was met: PT‐INR ≥ 1.5, PT activity ≤ 40%, or T‐Bil ≥ 5.0 mg/dL [4].

The disease severities of patients were also classified into grades 0, 1a, 1b, 2, 3a, and 3b using the chronic liver failure consortium organ failure (CLIF‐C OF) score, according to the existing diagnostic criteria [18].

#### 2.6.3 Handling of Anticoagulation and PT Activity (%)

In patients receiving anticoagulants where PT activity could not be determined, we did not exclude these cases. For baseline Child‐Pugh assignment, the coagulation item was calculated under both a 1‐point (best case) and a 3‐point (worst case) scenario. Only patients whose total Child‐Pugh score remained 5–9 in both scenarios were eligible for ACLF classification; others were not classified as ACLF (while remaining within the screened cirrhosis cohort).

#### 2.6.4 Infection

Infection was defined as a clinically suspected focus with microbiological evidence and/or imaging, requiring targeted, antimicrobial therapy.

### 2.7 Population

The study population comprised 363 patients with liver cirrhosis (Figure S1). Within this cohort, 10 patients (2.8%) had confirmed ACLF, 30 (8.3%) had extended ACLF, and 323 (89.0%) were non‐ACLF. A total of 40 patients with either confirmed or extended ACLF were included as the target population for further clinical and prognostic analyses. We plotted Kaplan–Meier curves across two groups (confirmed ACLF and extended ACLF).

### 2.8 Outcomes and Measures

Primary outcome was time to all‐cause death within 90 days from ACLF onset, with administrative censoring at day 90 [15].

### 2.9 Statistical Analysis

Descriptive statistics were used to summarize the baseline characteristics of the study population. Continuous variables are presented as means with standard deviations, and categorical variables as frequencies with percentages. Continuous factors were dichotomized at their median values. We fitted Cox proportional hazards models with a pre‐specified, parsimonious covariate set: age (per 10 years) and MELD‐Na (per 5 points). Beyond the pre‐specified two‐variable Cox model (age per 10 years; MELD‐Na per 5 points), we fitted three sensitivity Cox models, each adding one clinically meaningful binary covariate to avoid overfitting: precipitating infection (yes/no), gastrointestinal bleeding (yes/no), or alcohol as the precipitating factor (yes/no). Estimates are summarized to enhance interpretability; the primary inference remains based on the continuous‐variable model. Statistical significance was set at *p* < 0.05. All data were analyzed using SPSS Statistics software version 24 (IBM Corp., Armonk, NY, USA).

### 2.10 Data Management

Data management practices included regular audits to ensure accuracy and completeness. Any discrepancies were resolved by cross‐referencing the original patient records.

## 3 Results

**FIGURE 1:**
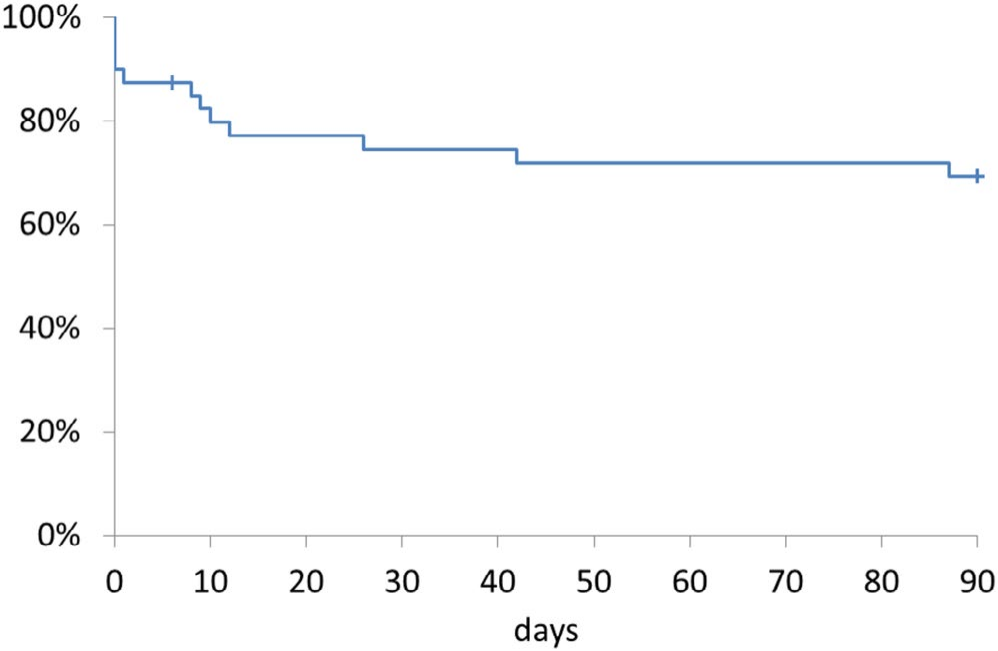
Kaplan–Meier survival to day 90 in ACLF (confirmed and extended).

**FIGURE 2:**
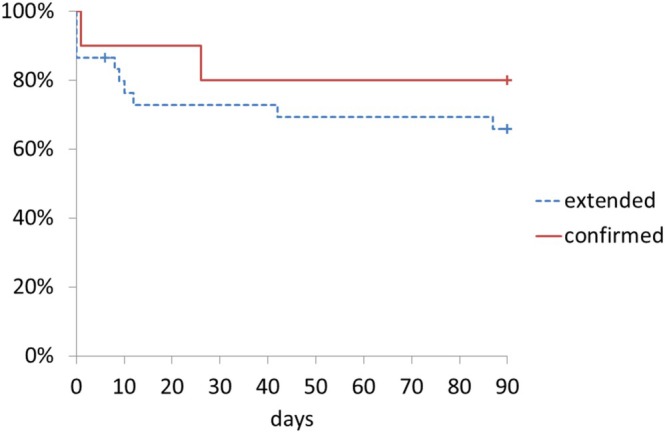
Kaplan–Meier survival to day 90 in confirmed versus extended ACLF.

This retrospective study analyzed 363 patients with liver cirrhosis who were hospitalized at the Study center between April 2014 and December 2022 to determine the incidence, clinical characteristics, treatment outcomes, and prognosis of ACLF (Figure S1). No patients were excluded. ACLF was diagnosed in 40 patients (11.0%) (10 confirmed cases (2.8%) and 30 extended cases (8.3%)). The 90‐day mortality by Kaplan–Meier was 30.5% (Figure 1). Between the two ACLF categories, 90‐day mortality did not differ significantly (confirmed 20.0% vs. extended 34.1%; log‐rank *p* = 0.42; Figure 2). As pre‐specified, these findings support analyzing confirmed and extended ACLF as a combined cohort under the Japanese framework; however, given the small sample and event counts, they should not be taken to imply equivalence between categories.

Among the 40 patients with ACLF, the mean age was 64.4 ± 13.6 years and the median age was 65.5 years (Table 1). All patients were Asian (Japanese). The cohort comprised 27 men and 13 women. The etiologies of cirrhosis among the patients exhibited the following variations: Twenty cases were attributed to alcohol consumption alone; six cases were related to chronic hepatitis C virus (HCV) infection for which direct‐acting antiviral (DAA) therapy had not yet been administered; one case involved combined alcohol consumption and untreated HCV infection; three cases were linked to resolved hepatitis B virus (HBV) infection (past‐infection pattern); one case combined alcohol consumption with HBV infection that was being treated with nucleos(t)ide analogue therapy; three cases were due to non‐alcoholic steatohepatitis; three to autoimmune hepatitis; and three remained of unknown etiology. Before ACLF onset, Child–Pugh scores ranged from 5 to 9: six patients scored 5, five scored 6, five scored 7, seven scored 8, and 10 scored 9. In anticoagulated patients with unavailable PT activity, we included cases only when baseline Child‐Pugh remained 5–9, as pre‐specified in Methods (*n* = 7). Factors contributing to acute exacerbations included gastrointestinal bleeding (*n* = 8), gastrointestinal bleeding + alcohol consumption (*n* = 6), gastrointestinal bleeding + infection (*n* = 1), alcohol consumption (*n* = 7), alcohol consumption + infection (*n* = 1), alcohol consumption + other (*n* = 1), infection (*n* = 11), and others (*n* = 5) (Categories were not mutually exclusive; overlaps were allowed.). The mean Model for End‐Stage Liver Disease Sodium (MELD‐Na) score at the time of onset was 20.7 (±7.5), with a median of 20.5 [16]. The severity of ACLF was classified as follows: 0 points in 26 cases, 1 point in six cases, 2 points in four cases, and 3 points in four cases. Treatment included corticosteroid administration in four patients (10.0%). Regarding the CLIF‐C OF scores, the distribution was as follows: 25, 5, 4, 3, 2, and 1 patient had scores of 0, 1a, 1b, 2, 3a, and 3b, respectively. None of the patients received artificial liver support therapy, granulocyte‐monocyte apheresis therapy, or liver transplantation.

**TABLE 1:** Demographic and clinical characteristics of the 40 patients with ACLF.

Feature	Value	Distribution
Age (years)	64.4 ± 13.6	Median 65.5
Sex	Male	27 cases (67.5%)
	Female	13 cases (32.5%)
Child‐Pugh score (prior to onset)	5 points	6 cases (15.0%)
	6 points	5 cases (12.5%)
	7 points	5 cases (12.5%)
	8 points	7 cases (17.5%)
	9 points	10 cases (25.0%)
	Unclear, but between 5–9	7 cases (17.5%)
Cirrhosis etiology (duplicate data included)	Alcohol	22 cases (55.0%)
	HCV	7 cases (17.5%)
	HBV	4 cases (10.0%)
	NASH	3 cases (7.5%)
	AIH	3 cases (7.5%)
	Unknown	3 cases (7.5%)
Acute exacerbation factors (duplicate data included)	Gastrointestinal bleeding	17 cases (42.5%)
	Alcohol consumption	15 cases (37.5%)
	Infection	13 cases (32.5%)
	Other	6 cases (15.0%)
MELD‐Na score	20.7 ± 7.5	Median 20.5
CLIF‐C OF score	0	25 cases (62.5%)
	1a	5 cases (12.5%)
	1b	4 cases (10.0%)
	2	3 cases (7.5%)
	3a	2 cases (5.0%)
	3b	1 case (2.5%)
ACLF severity	0	26 cases (65.0%)
	1	6 cases (15.0%)
	2	4 cases (10.0%)
	3	4 cases (10.0%)
Treatment	Adrenal cortex hormones	4 cases (10.0%)
	Artificial liver support therapy	0 cases (0%)
	Granulocyte/monocyte apheresis	0 cases (0%)
	Liver transplant	0 cases (0%)

As pre‐specified, the primary analysis used Cox regression for time to 90‐day all‐cause death. In the Cox model, age (per 10 years) was associated with higher 90‐day mortality (HR, 2.29; 95% CI 1.31–4.00; *p* < 0.01), as was MELD‐Na (per 5 points) (HR, 1.74; 95% CI 1.16–2.63; *p* < 0.01) (Table 2). When infection, gastrointestinal bleeding, or alcohol was added one at a time to age and MELD‐Na, the hazard ratios for age and MELD‐Na remained directionally consistent with the primary model and did not materially change. The added covariates showed imprecise, non‐significant associations and were not retained as independent predictors within our sample. For bedside interpretability, Table 3 cross‐classifies age (< 66/≥ 66) and MELD‐Na (< 21/≥ 21) with Kaplan–Meier 90‐day mortality (95% CIs) and the number of patients in each stratum (n).

**TABLE 2:** Cox proportional hazards for 90‐day all‐cause death (primary model).

Variable	HR (95% CI)	*p*
Age (per 10 years)	2.29 (1.31–4.00)	< 0.01
MELD‐Na (per 5 points)	1.74 (1.16–2.63)	< 0.01

**TABLE 3:** Ninety‐day mortality in the combined ACLF cohort by age and MELD‐Na categories (display only; primary analysis uses continuous predictors).

	MELD‐Na < 21	MELD‐Na ≥ 21
Age < 66 years	*n* = 8 Kaplan–Meier 90‐day mortality: 0% [95% CI 0%–0%]	*n* = 12 Kaplan–Meier 90‐day mortality: 17.5% [95% CI 0%–39.6%]
Age ≥ 66 years	*n* = 12 Kaplan–Meier 90‐day mortality: 25.0% [95% CI 0.5%–49.5%]	*n* = 8 Kaplan–Meier 90‐day mortality: 87.5% [95% CI 64.6%–100%]

*Note:* Percentages are Kaplan–Meier (KM) estimates at day 90 with 95% confidence intervals. Dichotomized thresholds were not used for variable selection; the primary Cox model used age (per 10 years) and MELD‐Na (per 5 points) as continuous predictors.

## 4 Discussion

In our study, the incidence of ACLF among hospitalized patients with cirrhosis was 2.8% in the confirmed cases and 8.3% in the extended cases. Thus, its prevalence is significant and should not be ignored. Because our classification adhered to the Japanese ACLF criteria (requiring baseline Child‐Pugh 5–9), the observed incidence pertains to this definition and may differ from EASL CLIF‐based estimates. The significant incidence observed in our cohort highlights the need for broad and inclusive diagnostic approaches to accurately determine the true burden of ACLF in clinical settings.

Gastrointestinal bleeding, alcohol consumption, and infection have been identified as the primary acute exacerbation factors associated with ACLF. These findings align with the existing literature which shows the significant impact of these factors on liver disease progression and acute decompensation [3, 17]. This emphasizes the need for proactive management strategies targeting these exacerbating factors to prevent the onset of ACLF. Early identification and management of gastrointestinal bleeding and infections, coupled with effective counseling and interventions for alcohol use, are crucial for mitigating the risk of ACLF development. In addition to these measures, enhanced nutritional support and regular monitoring of patients for early signs of decompensation could be considered to further reduce the risk of ACLF development.

We pre‐specified a two‐variable Cox model (age, MELD‐Na) to avoid overfitting given limited events and to maximize clinical interpretability. Under the Japanese criteria, baseline Child–Pugh is restricted to 5–9, limiting variability and potentially overlapping with MELD‐Na; thus Child–Pugh was not included in the primary model. Using a pre‐specified, parsimonious Cox model, we found that older age (per 10 years) and MELD‐Na (per 5 points) were independently associated with a higher hazard of death within 90 days. In exploratory sensitivity analyses, adding single clinically meaningful precipitants (infection, gastrointestinal bleeding, alcohol) one at a time did not improve model performance and did not alter the interpretation of age and MELD‐Na. This likely reflects limited power and heterogeneity of precipitants rather than a lack of clinical importance. Under our events‐per‐variable constraint, age and MELD‐Na appear to capture most of the short‐term prognostic signal; these sensitivity analyses therefore support the robustness and applicability of the primary findings. These findings are consistent with those of previous studies highlighting the critical role of these factors in determining patient outcomes [8, 11]. The MELD‐Na score is a well‐established prognostic tool which reflects both the severity of liver disease and the presence of hyponatremia, a common and severe complication of cirrhosis [9]. Older age, a significant predictor of poor prognosis, underscores the need for tailored management approaches in older patients with ACLF. Age‐related physiological changes and comorbidities may complicate the clinical course and response to treatment in older patients. Therefore, comprehensive geriatric assessment and individualized care plans are essential to improve outcomes in this vulnerable population.

Among the 40 ACLF cases in this study, consideration for deceased‐donor liver transplantation was evaluated based on age and the duration of alcohol abstinence (Figure S2). An analysis of national registry data, including both deceased and living donor liver transplants, showed that harmful relapse to alcohol use after liver transplantation was significantly less common in patients with ≥ 18 months of abstinence (2.9% vs. 15.6%) [19]. In Japan, the period of abstinence required before liver transplantation for alcoholic liver disease is 18 months for deceased‐donor liver transplantation. Twenty of the patients in this study were aged 65 years or above, making them ineligible for transplantation. Regarding the other 20 patients, 12 had abstained from alcohol for less than 18 months and were also ineligible. Of the eight remaining patients, six survived and two died. Two patients had uncontrolled infections, further precluding transplantation eligibility. Therefore, none of the patients in our study was eligible for liver transplantation, reflecting a significant clinical challenge in managing ACLF. This limited intervention reflects the challenges in managing ACLF within the current therapeutic landscape [2, 3]. Specifically, the low eligibility rate for transplantation underscores the limitations of current treatment options and the necessity for innovations in both medical and supportive care for patients with ACLF, including the development of enhanced internal medical care strategies.

The results of this study also underscore the high mortality associated with ACLF and the progressive decline in survival over time, corroborating the findings of previous studies [8, 12]. This high mortality rate indicates a poor prognosis for ACLF and emphasizes the urgent need for effective management strategies to improve patient outcomes. To minimize omissions, anticoagulated patients with unavailable PT activity were retained, using a conservative pre‐specified approach in which baseline Child‐Pugh was assessed under both best‐ and worst‐case coagulation scoring and verified to remain 5–9 (see Methods).

Regarding the national data, 72 confirmed ACLF cases were registered at 799 facilities nationwide in 2020 (Table 4). Based on the frequency observed in our department, it is estimated that there are actually 913 nationwide confirmed cases annually. This suggests that only 7.9% of actual ACLF cases were registered in the national tally. The national estimate derived from our center's frequency is exploratory and intended to frame hypothesis generation, not to represent actual counts. Regarding the potential underreporting of ACLF cases, cases registered in the national statistics primarily represent those from university hospitals and advanced medical institutions, which probably account for only a portion of patients receiving multidisciplinary treatment. In contrast, many more patients are treated at city hospitals, where the reported data and papers on prognosis may diverge significantly from actual clinical realities. Our study, conducted at a hospital with minimal demographic changes, aimed to provide a clear picture with less patient selection bias, reflecting a more representative patient cohort and enhancing the validity of our findings. Real‐world data can provide insights into the natural history of ACLF, effectiveness of different treatment modalities, and impact of various prognostic factors in a more diverse and representative patient cohort [10]. Such data can help bridge the gap between clinical research and practice, leading to more effective and personalized treatment approaches. However, generalizability beyond similar settings is limited by referral patterns and local practice.

**TABLE 4:** Comparison of confirmed ACLF cases in the study center and National Data.

Duration	The study center (number of observed cases)	All 799 facilities nationwide (estimates)	All 799 facilities nationwide (number of registered cases)
8 years 9 months	10 cases	7990 cases	
1 year	1.14 cases	913 cases	72 cases (7.9%)

Nakayama et al. and Mochida et al. emphasized the need for accurate diagnostic criteria and comprehensive data collection to understand ACLF [4, 6, 14, 15]. The diagnostic criteria for ACLF and related disease conditions in Japan, proposed by these researchers, provide a framework for identifying and managing ACLF in clinical practice. These criteria are crucial for standardizing diagnosis and improving the accuracy of ACLF data collection, which can subsequently inform better treatment strategies and policy‐making [14].

## 5 Limitations

The retrospective design and single‐center data limit the generalizability of the findings. Although our study provides valuable insights into the incidence and predictors of ACLF, prospective multicenter studies are needed to validate these findings and expand our understanding of ACLF in diverse clinical settings. Additionally, the reliance on electronic health records and patient charts for data collection may introduce information bias, although efforts have been made to ensure data accuracy through regular audits and cross‐referencing with original records. We acknowledge that the number of confirmed ACLF cases (*n* = 10) was small and may be insufficient for robust comparative survival analysis. While the small sample size limits statistical power, we believe that even a modest number of rigorously defined real‐world cases offers important insight into clinical patterns and management gaps that warrant further prospective validation.

## 6 Conclusions

This study underscores the importance of comprehensive management strategies targeting common exacerbation factors and key prognostic indicators in patients with ACLF. This observational study does not evaluate treatment effects. We therefore refrain from prescriptive recommendations. Our findings—particularly the prognostic impact of age and MELD‐Na—support risk stratification and timely management within standard care pathways (e.g., early evaluation for infection or bleeding as clinically indicated) but should not be interpreted as evidence of efficacy for specific interventions. Future research should focus on prospective multicenter studies to validate these findings and further refine ACLF management strategies.

## Ethics Statement

This study was conducted in accordance with the principles outlined in the Declaration of Helsinki. It was approved by the Ethics Committee of the Eastern Chiba Medical Center (approval no. 80).

## Consent

Owing to the retrospective design of the study, the requirement for informed consent from participants was waived. This waiver was granted by the Ethics Committee of the Eastern Chiba Medical Center in accordance with institutional guidelines and regulations.

## Conflicts of Interest

The authors declare no conflicts of interest.

## Supporting information


**Figure S1:** Patient distribution.

**Figure S2:** Indications for cadaveric liver transplantation.

## Data Availability

The data that support the findings of this study are available from the corresponding author upon reasonable request.
