# Supplementary figures and images for: Incidence and Short‐Term Prognosis of Acute‐On‐Chronic Liver Failure Defined by Japanese Criteria: A Single‐Center Retrospective Cohort Study From Urban Japan
Source: JGH Open. 2025 Nov 10;9(11):e70300. doi: 10.1002/jgh3.70300 (PMC12602991; doi:10.1002/jgh3.70300)

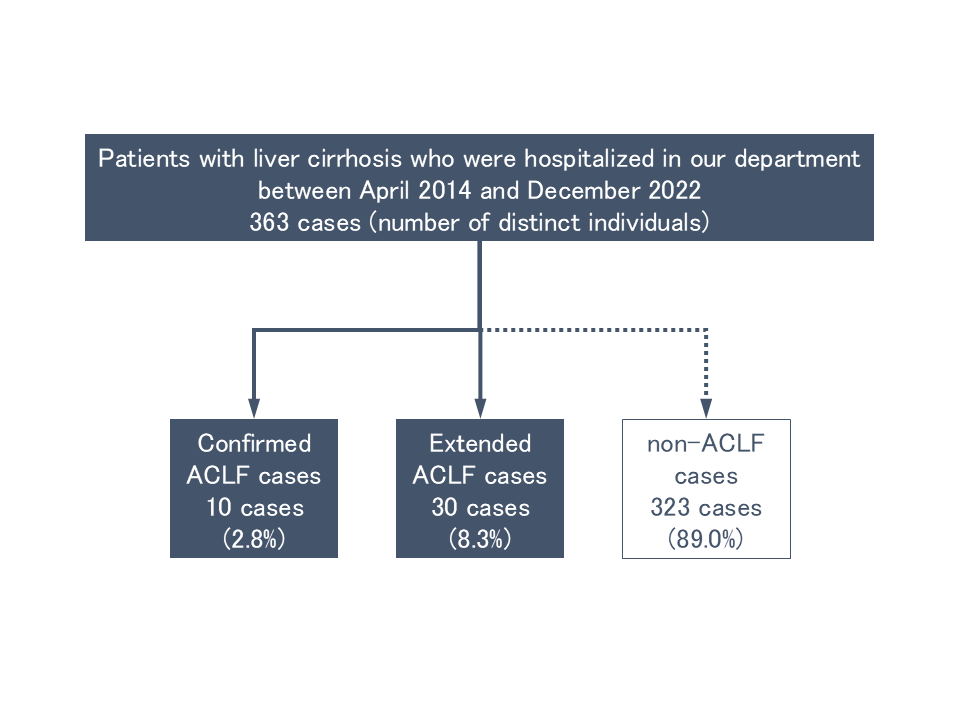

Supplement: Supplementary file 1 — Figure S1: Patient distribution. [file JGH3-9-e70300-s002.tif]

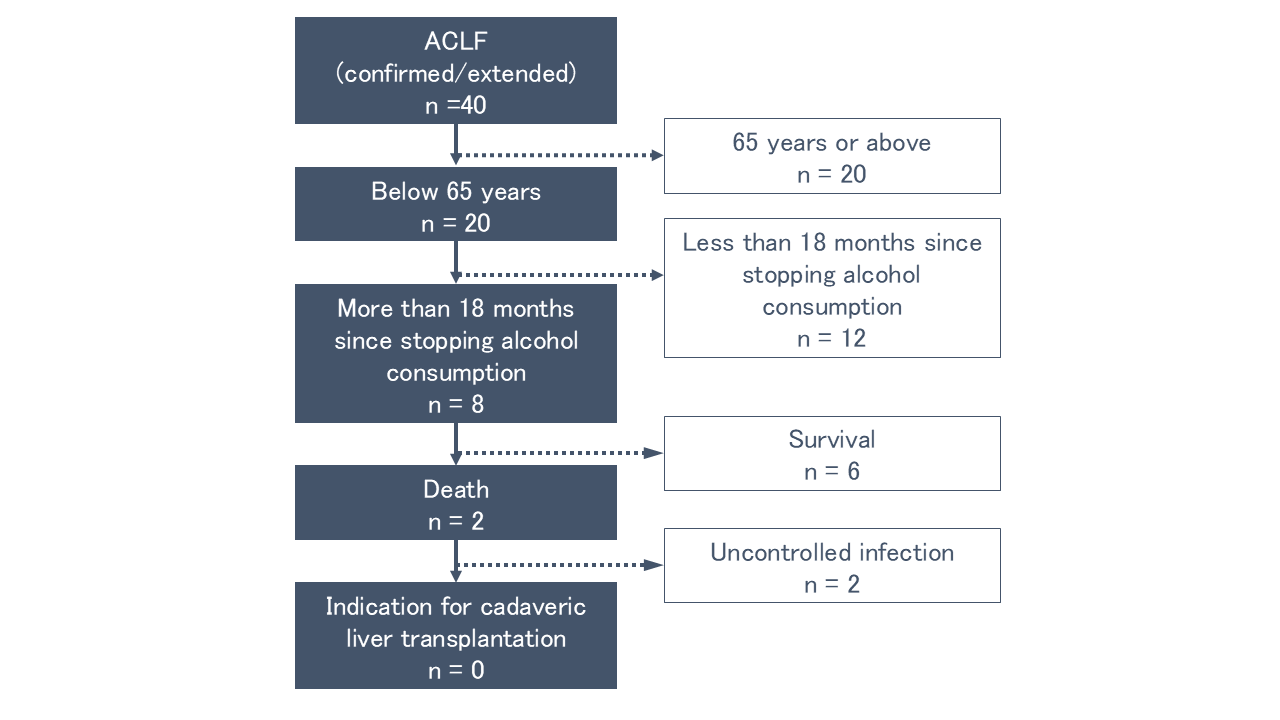

Supplement: Supplementary file 2 — Figure S2: Indications for cadaveric liver transplantation. [file JGH3-9-e70300-s001.tif]
